# 

**Published:** 1804-01-01

**Authors:** 


### MEDICAL

AND

### PHYSICAL

### JOURNAL.

CONDUCTED BY

T. BRADLEY, M.D.

R. BATTY, M.D.

AND

A. A. NOEHDEN, M.D.

VOL. XI.

FROM JANUARY TO JUNE, 1804.

*LONDON:*

PRINTED FOR RICHARD PHILLIPS, NO. 71, ST. PAUL'S CHURCH YARD.

[ By William Thorne, Red Lion Court, Fleet Street. ]

### TO CORRESPONDENTS.

S. J's. Paper on the Means of preventing Contagion will appear in our next Number.

Philomedicus is postponed for want of a proper Signature.

Communications are received from Dr. Aberdour, Dr. Scott, Mr. Marson, Mr. Boswell, Mr. Ward, and Mr. G. N. Hill.

